# *Bcl-xL* Silencing Induces Alterations in hsa-miR-608 Expression and Subsequent Cell Death in A549 and SK-LU1 Human Lung Adenocarcinoma Cells

**DOI:** 10.1371/journal.pone.0081735

**Published:** 2013-12-10

**Authors:** Norahayu Othman, Lionel L. A. In, Jennifer A. Harikrishna, Noor Hasima

**Affiliations:** 1 Institute of Biological Sciences, Division of Genetics and Molecular Biology, Faculty of Science, University of Malaya, Kuala Lumpur, Wilayah Persekutuan, Malaysia; 2 Centre for Research in Biotechnology for Agriculture (CEBAR), University of Malaya, Kuala Lumpur, Wilayah Persekutuan, Malaysia; IPMC, CNRS UMR 7275 UNS, France

## Abstract

Bcl-xL is an anti-apoptotic protein that is frequently found to be overexpressed in non-small cell lung cancer leading to an inhibition of apoptosis and poor prognosis. Recently, the role of miRNAs in regulating apoptosis and cell survival during tumorigenesis has become evident, with cancer cells showing perturbed expression of various miRNAs. In this study, we utilized miRNA microarrays to determine if miRNA dysregulation in *bcl-xL* silenced lung adenocarcinoma cells could be involved in regulating cell death. Short interfering RNA-based transfection of A549 and SK-LU1 lung adenocarcinoma cells was successful in inducing a reduction in *bcl-xL* expression levels, resulting in a decrease in cell viability. A total of 10 miRNAs were found to be significantly differentially expressed when compared between siRNA-transfected and non-transfected cells including hsa-miR-181a, hsa-miR-769-5p, hsa-miR-361-5p, hsa-miR-1304 and hsa-miR-608. When overexpression studies on hsa-miR-608 was performed via transfection of miRNA mimics, cell death was found to be induced in A549 and SK-LU1 cells in comparison to untreated cells. This effect was reversed when knockdown studies involving anti-sense inhibitors were introduced. Combination of siRNA based silencing of *bcl-xL* (si*Bcl-xL*) followed by anti-sense inhibitor transfection led to a decrease in the apoptotic population of A549 and SK-LU1 cells in comparison to cells only treated with si*Bcl-xL*, illustrating the connection between *bcl-xL*, hsa-miR-608 and cell death. Gene target prediction analysis implicated the PI3K/AKT, WNT, TGF-β, and ERK signaling pathways as targets of *bcl-xL* induced miRNA alterations. We have demonstrated that *bcl-xL* silencing in A549 and SK-LU1 cells leads to the occurrence of cell death through the dysregulation of specific miRNAs. This study also provides a platform for anti-sense gene therapy whereby miRNA expression can be exploited to increase the apoptotic properties in lung adenocarcinoma cells.

## Introduction

In contrast to normal cells, cancer cells have the ability to disrupt the balance between pro and anti-apoptotic factors to promote cell survival under the conditions of environmental stress. In terms of molecular events occurring in tumors, evasion of apoptosis is an important hallmark of tumor progression, where members of the evolutionarily conserved B-cell lymphocyte 2 (Bcl-2) family are thought to be the central regulators [1]. The expression level of *bcl-2* differs between various cell types, however high levels and aberrant patterns of *bcl-*2 expression have been reported in a wide variety of human cancers [2]. Elevation of Bcl-2 protein expression contributes not only to the development of cancer but also to resistance against a wide variety of anti-cancer agents [3]–[5]. However, studies conducted on non-small cell lung cancers (NSCLCs), which accounts for the majority of lung cancer cases [6], have shown that the expression of Bcl-2 is either very low or absent [1]. Instead, the expression of B-cell lymphocyte xL (*bcl-xL*), the other major prototype of the anti-apoptotic *bcl-2* gene, is shown to be overexpressed in NSCLCs [7]. Over-expression of Bcl-xL has been shown to counteract the pro-apoptotic functions of Bcl-2 associated X protein (Bax) and Bcl-2-associated death promoter (Bad) by preventing their translocation from the cytosol to the mitochondria. This inhibits apoptosis by maintaining the permeability status or stabilization of the outer mitochondrial membrane, which subsequently prevents cytochrome c release and pro-caspase-9 activation [8].

MicroRNAs (miRNAs) are small non-coding RNAs of about 19 to 23 nucleotides long that regulate gene expression post-transcriptionally, by either inhibiting mRNA translation or by inducing mRNA degradation [9]. These regulatory elements play a role in a wide range of biological processes including cell proliferation, differentiation and apoptosis [10]–[12]. Therefore, alterations in miRNA function and expression may disorganize cellular processes and eventually cause or contribute to disease progression, including cancer [13]. For example, recent studies have shown that miR-133 acts as a regulator of survival in cardiac cells by repressing caspase-9 expression at both protein and mRNA levels [14], while the miR-17-92 cluster, which is amplified in B cell lymphomas, is capable of inhibiting apoptosis by negatively regulating the tumor suppressor PTEN and the pro-apoptotic protein B-cell lymphocyte 11 (Bim) [15]. While many miRNAs have been identified to be dysregulated in cancers, their specific functions remain unclear due to the nonspecific binding properties of each individual miRNA. As the miRNA field continues to evolve and develop, it is important to gain a better understanding of miRNA biogenesis and function, as it will certainly affect the development of miRNA-based therapies.

Therefore, this study describes the siRNA-based silencing of the anti-apoptotic *bcl-xL* gene, followed by the establishment of a global miRNA expression profile through the comparison between silenced and non-silenced cells. We hypothesized that *bcl-xL* silencing in A549 cells would result in different miRNA expression patterns which could potentially be used for anti-sense gene therapeutic applications in NSCLC.

## Methods

### 2.1 Cell Lines and Culture Conditions

Human lung adenocarcinoma cell line (A549) and normal human nasopharyngeal epithelial cell line (NP-69) were obtained from Cancer Research Initiative Foundation (CARIF), Sime Darby Medical Centre, Malaysia. Human lung adenocarcinoma cell line (SK-LU1) was purchased from AseaCyte Sdn. Bhd., Malaysia. A549 cells were cultured in Roswell Park Memorial Institute 1640 (RPMI-1640) (Thermo Scientific Hyclone, USA) culture medium, supplemented with 10% (v/v) heat inactivated fetal bovine serum (FBS) (JR Scientific Inc., USA) while SK-LU1 cells were cultured in minimum essential medium alpha (MEM-α) (Life Technologies, USA), supplemented with 10% (v/v) heat inactivated FBS (JR Scientific Inc., USA). NP-69 cells were cultured in keratinocyte serum-free medium (KSFM) (Gibco, USA) supplemented with 1×2.5 µg human recombinant epidermal growth factor (rEGF) (Gibco, USA) and 1×2.5 mg bovine pituitary extract (Gibco, USA). All cells were grown as a monolayer and maintained in 95.0% relative humidity and 5.0% CO_2_ levels at 37.0°C.

### 2.2 Transfection of siRNA

Stealth™ RNAi siRNA Duplex Oligonucleotides were purchased from Invitrogen, USA as follows: BCL2L1-HSS141361 (5′-UCACUAAACUGACUCCAGCUGUAUC-3′), BCL2L1-HSS141362 (5′-CCCAGUGCCAUCAAU GGCAAC CCAU-3′) and BCL2L1-HSS141363 (5′-GCAGUUUGGAUGCCCGGGAGGUGAU-3′). Stealth™ RNAi Negative Control Duplex (Invitrogen, USA) with low GC content and high GC content was used as negative scramble RNA controls for the RNAi response. For each set of experimental siRNA, two flasks of cells were plated concurrently for 24 h prior to transfection. One flask was transfected with 100.0 nM Stealth™ RNAi complexed with 2.0 µg/ml Lipofectamine™ 2000 (Invitrogen, USA), while the other was transfected with 100.0 nM BLOCK-iT™ Alexa Fluor® Red Fluorescent Oligo (Invitrogen, USA) complexed with 2.0 µg/ml Lipofectamine™ 2000 according to manufacturer’s protocol. After 24 h, transfection efficiency was assessed by visualizing uptake of BLOCK-iT™ Alexa Fluor® Red Fluorescent Oligo using fluorescence microscopy. At 24 h after transfection, total RNA was isolated using TRIzol® Reagent (Invitrogen, USA) and analyzed using the ScreenTape R6K on the Agilent 2200 TapeStation (Agilent Technologies, Germany), according to the manufacturer’s protocol.

### 2.3 MTT Cell Viability Assay

Cytotoxic effects of *bcl-xL* silenced NP-69, A549 and SK-LU1 cells were determined using MTT assay according to previously described protocol [16]. Briefly, 1.0×10^4^ cells/well were plated, incubated overnight and transfected with siRNA. Following incubation, 20.0 µl MTT reagent (5.0 mg/ml) was added into each well and incubated in the dark at 37°C for 2 h. Media containing excess MTT reagent was aspirated and DMSO was added to dissolve purple formazan precipitates. Results were obtained using a microtiter plate reader (Tecan Sunrise®, Switzerland) at 570 nm absorbance wavelength and 650 nm reference wavelength.

### 2.4 Annexin V-FITC Apoptosis Assay

The presence of apoptosis in *bcl-xL* silenced cells was detected using the annexin V-FITC apoptosis detection kit (Calbiochem, USA) according to the manufacturer’s protocol. Briefly, 500 µl cold 1× binding buffer containing 2.0 µl annexin V-FITC was added to siRNA-transfected and non-transfected cells, followed by 15 min incubation at 27°C in the dark. All samples were centrifuged at 1000×g for 5 min and re-suspended in 500 µl cold 1× binding buffer with 10.0 µl of propidium iodide. Detection of signals from a 1.0×10^4^ cell population was obtained using the BD FACSCanto™ II flow cytometer (BD Biosciences, USA) and examined on the BD FACSDiva™ (BD Biosciences, USA) software.

### 2.5 Quantitative Reverse Transcription Polymerase Chain Reaction (RT-qPCR)

RT-qPCR analysis on equivalent amounts of mRNA was performed using the SuperScript™ III Platinum® SYBR® Green Two-Step RT-qPCR Kit with ROX according to the manufacturer’s protocol (Invitrogen, USA). Primers used for qPCR reactions are as follows: Bcl-xL forward (5′-CGTGGAAAGCGTAGACAAGGA-3′) and reverse (5′-ATTCAGG TAAGTGGCCATCCAA-3′), β-actin forward (5′-AAGCCACCCCACTTC TCTCTAA-3′) and reverse (5′-ACCTCCCCTGTGTGGACTTG-3′). RT reactions contained 1.0 µg of total RNA samples, 10.0 µl 2× RT reaction mix, 2.0 µl enzyme mix, and topped up to 20.0 µl with DEPC treated water. The thermal profile for RT consisted of incubation at 42°C for 50 min and termination of the reaction at 85°C at 5 min. 1.0 µl (2.0 U/µl) of *E. coli* RNase H was added and incubated at 37°C for 20 min. 5.0 µl of undiluted cDNA was used in the qPCR protocol. qPCR reactions contained 25.0 µl Platinum® SYBR® Green qPCR SuperMix-UDG with ROX, 1.0 µl forward primer (10.0 µM), 1.0 µl reverse primer (10.0 µM), and topped up to 50.0 µl with DEPC-treated water. Real-time PCR was performed using the CFX96™ Real-Time PCR Detection System (Bio-Rad Laboratories, USA) and analyzed using the Bio-Rad CFX Manager™ v1.6 (Bio-Rad Laboratories, USA). Reactions were performed in triplicates for each sample. The PCR thermal profile consisted of activation steps (50°C for 2 min, 90°C for 2 min), followed by 40 cycles of denaturation at 95°C for 15 sec, annealing and extension at 60°C for 30 sec and subsequent melting curve analysis. For each sample, expression of target genes was normalized to the expression of β-actin. Fold change was calculated using the Pfaffl method [17], and presented as fold change in expression relative to non-transfected controls.

### 2.6 Western Blotting

Protein was extracted following 48 h of transfection using the NE-PER® Nuclear and Cytoplasmic Extraction Kit (Pierce, USA) according to manufacturer’s protocol. Size separation by electrophoresis in 12% (w/v) SDS-PAGE was performed before transfer to nitrocellulose membranes. Membranes containing Bcl-xL proteins were blocked with 1× Tris-buffered saline (TBS), plus 5.0% (w/v) non-fat skim milk powder and 0.05% (v/v) Tween-20 (Promega, USA), while membranes containing GAPDH proteins were blocked with 1× TBS, plus 5.0% bovine serum albumin (BSA) (Calbiochem, USA) and 0.05% Tween20. Membranes were incubated at 4°C overnight with primary antibodies: Bcl-xL rabbit monoclonal antibody and GAPDH rabbit monoclonal antibody (Cell Signaling Technology, USA). Blots were washed with 1× Tris buffered saline-Tween20 (TBST) buffer, incubated with secondary anti-rabbit IgG HRP-linked antibody and anti-biotin HRP-linked antibody (Cell Signaling Technology, USA), and washed again with 1× TBST and 1× TBS buffer. Bands were visualized using the SuperSignal® West Pico Chemiluminescent Substrate (Thermo Scientific, USA) on the Fusion FX7 system (VilberLourmat, France) and quantified using the ImageJ Analyst software (NIH, USA), with band intensities normalized to GAPDH.

### 2.7 MiRNA Microarray

MiRNA microarray was carried out on the A549 cells using the FlashTag™ Biotin RNA Labeling Kit for Affymetrix® GeneChip®1.0 miRNA Arrays (Genisphere, USA) and GeneChip® miRNA Array (Affymetrix, USA) according to the manufacturer’s protocol. Briefly, 1.0 µg of total RNA was labeled and hybridized on to the miRNA microarray chips containing 46,228 probes representing over 6,703 miRNA sequences from 71 organisms from the Sanger miRNA database v11. GeneChip® miRNA arrays were scanned using GeneChip® Scanner 3000 7G (Affymetrix, USA) and digitized using GeneChip® Operating Software (GCOS) (Affymetrix, USA) and GeneChip® Command Console® Software (AGCC) (Affymetrix, USA) v3.0.1. Raw data quality control was conducted using the miRNA QC Tool (Affymetrix, USA) v1.0.33.0, while analysis of expression data was performed using the Partek® Genomics™ Suite (PGS) (Partek Incorporated, USA) software. Background correction and probe set summarization was done using the Robust Multichip Averaging (RMA) method. Statistical analysis was performed using the Analysis of Variance (ANOVA) approach. Differentially expressed miRNAs were statistically analyzed using ANOVA with a *p*-value threshold of ≤0.05, and filtered based on a ≥1.5 fold change threshold between siRNA-transfected and non-transfected samples. All microarray data has been deposited in NCBI’s Gene Expression Omnibus [18] and is accessible through GEO Series accession number: GSE47059 at http://www.ncbi.nlm.nih.gov/geo/query/acc.cgi?acc=GSE47059.

### 2.8 Quantification of miRNA Expression

RT-qPCR analysis of miRNA expression was carried out using TaqMan® MicroRNA Assays (Applied Biosystems, USA) according to the manufacturer’s protocol. RT reactions contained 5.0 ng of total RNA samples, 2.0 µl stem-loop RT primer, 1.0 µl RT buffer, 0.1 µl dNTP (100.0 mM), 0.67 µl MultiScribe™ Reverse Transcriptase (50.0 U/µl) and 0.13 µl RNase inhibitor (20.0 U/µl). PCR reactions contained 0.67 µl RT product and 5.0 µl 2× TaqMan® 20× Primer Probe Assay. Reactions were performed in triplicate for each sample. The PCR thermal profile consisted of activation steps (50°C for 2 min, 95°C for 20 sec), 40 cycles of denaturation at 95°C for 3 sec, followed by annealing and extension at 60°C for 20 sec. qPCR was performed using the CFX96™ Real-Time PCR Detection System (Bio-Rad Laboratories, USA) and analyzed using the Bio-Rad CFX Manager™ v1.6 (Bio-Rad Laboratories, USA). Expression of target miRNAs was normalized to the expression of the U6 small nuclear RNA. Fold change was calculated using the 2^−ΔΔCt^ method [19], and presented as fold change expression relative to non-transfected controls.

### 2.9 Bioinformatics Analyses of miRNA Gene Targets

An *in silico* approach was used to identify the putative miRNA targets by using TargetScan Human v5.2 [20] and the database of conserved 3′UTR miRNA targets, found at http://www.targetscan.org/. Gene-annotation enrichment analyses of the predicted miRNA targets with total context scores of <0 were then performed using the web tool Database for Annotation, Visualization and Integrated Discovery (DAVID) v6.7 [21] at http://david.abcc.ncifcrf.gov/summary.jsp, using default parameters.

### 2.10 Transfection of microRNA Mimics and Hairpin Inhibitors

miRIDIAN microRNA mimics and hairpin inhibitors were purchased from Thermo Scientific, USA as follows: miRIDIAN microRNA human hsa-miR-608 mimic (AGGGGUGGUGUUGGGACAGCUCCGU) and miRIDIAN microRNA human hsa-miR-608 hairpin inhibitor (AGGGGUGGUGUUGGGACAGCUCCGU). miRIDIAN microRNA Mimic Negative Control #1 and miRIDIAN microRNA Hairpin Inhibitor Negative Control #1 (Thermo Scientific, USA) were used as negative scramble RNA controls for the RNAi response. miRIDIAN microRNA Mimic Transfection Control with Dy547 and miRIDIAN microRNA Hairpin Inhibitor Transfection Control with Dy547 (Thermo Scientific, USA) were used to determine transfection efficiency. Cells were plated for 24 h prior to transfection with miRIDIAN microRNA human hsa-miR-608 mimics or hairpin inhibitors complexed with DharmaFECT 1 Transfection Reagent (Thermo Scientific, USA) according to manufacturer’s protocol. After 24 h, transfection efficiencies were assessed by visualizing uptake of miRIDIAN MicroRNA Mimic/Hairpin Inhibitor Transfection Control with Dy547 using fluorescence microscopy. At 24 h after transfection, total RNA was isolated using TRIzol® Reagent (Invitrogen, USA) and analyzed using the ScreenTape R6K on the Agilent 2200 TapeStation (Agilent Technologies, Germany), according to the manufacturer’s protocol. RT-qPCR analysis of hsa-miR-608 expression was carried out using TaqMan® MicroRNA Assays (Applied Biosystems, USA) according to the manufacturer’s protocol. Expression of hsa-miR-608 was normalized to the expression of the U6 small nuclear RNA. The 2^−ΔΔCt^ method was used to determine relative quantitation of miRNA, and fold difference relative to scrambled negative controls was determined as Log2 (2^−ΔΔCt^). Cell death in mimic/hairpin inhibitor transfected cells was then detected using the Annexin V-FITC detection kit (Calbiochem, USA), according to the manufacturer’s protocol, 72 h post-transfection with miRIDIAN microRNA human hsa-miR-608 mimics/hairpin inhibitors.

### 2.11 Combined Transfection with si*Bcl-xL* and hsa-miR-608 Hairpin Inhibitors

To observe the connection between si*Bcl-xL*, hsa-miR-608 and cell death, cells were plated for 24 h prior to transfection with 100 nM Stealth™ RNAi (Invitrogen, USA): BCL2L1-HSS141361 (5′-UCACUAAACUGACUCCAGCUGUAU C-3′). 24 h post-transfection, spent media was removed and cells were transfected with miRIDIAN miRNA human hsa-miR-608 hairpin inhibitor (AGGGGUGGUGUUGGGACAGCUCCGU) (80 nM for A549, 20 nM for SK-LU1 and 20 nM for NP-69) or miRIDIAN miRNA Hairpin Inhibitor Negative Control #1 (Thermo Scientific, USA), which was used as negative scramble RNA controls for the RNAi response. Cell death was detected 72 h post-transfection using the Annexin V-FITC detection kit (Calbiochem, USA), according to manufacturer’s protocol.

### 2.12 Statistical Analysis

All experiments were performed in triplicate independent experiments. All data were presented as mean ± standard deviation (SD). Student’s t-test was used to determine the statistical significance of results, where a *p*-value of ≤0.05 was considered significant. Pearson’s correlation coefficient (*r*) value was used to determine the association between miRNA microarray and RT-qPCR data.

## Results

### 3.1 Silencing of *bcl-xL* Expression Resulted in a Reduction of A549 and SK-LU1 Cell Viability

**Figure 1:**
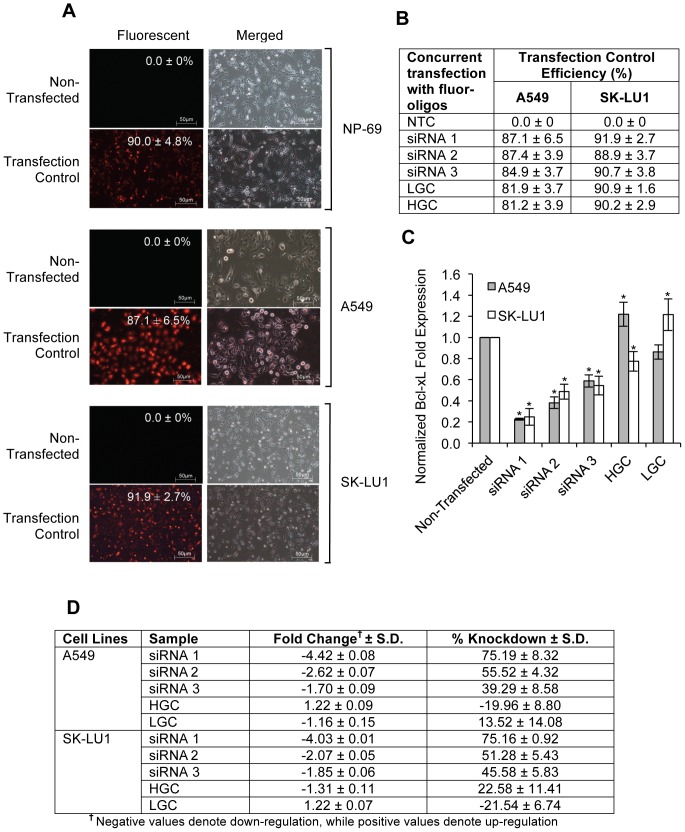
Silencing of *bcl-xL* using siRNA-based transfection. (**A**) Fluorescent and merged images of A549, SK-LU1 and NP-69 cells transfected with BLOCK-iT™ Alexa Fluor® Red Fluorescent Oligo. Percentage of mean transfection efficiency is indicated and all images shown are a representative of triplicates independent experiments. (**B**) Table indicating transfection control efficiency (%) in A549 and SK-LU1 cells. (**C**) RT-qPCR of normalized *bcl-xL* expression to endogenous *β-actin* expression in A549 and SK-LU1 cells. (**D**) Fold-change and knockdown of *bcl-xL* gene expression levels in siRNA-transfected cells in comparison to levels in non-transfected cells. All experiments were carried out in triplicates, and presented as mean ± S.D. Statistically significant differences in *bcl-xL* expression levels between non-transfected and transfected samples were indicated by *p-value ≤0.05. HGC denotes cells transfected with high GC content scramble RNA negative control. LGC denotes cells transfected with low GC content scramble RNA negative control.

**Figure 2:**
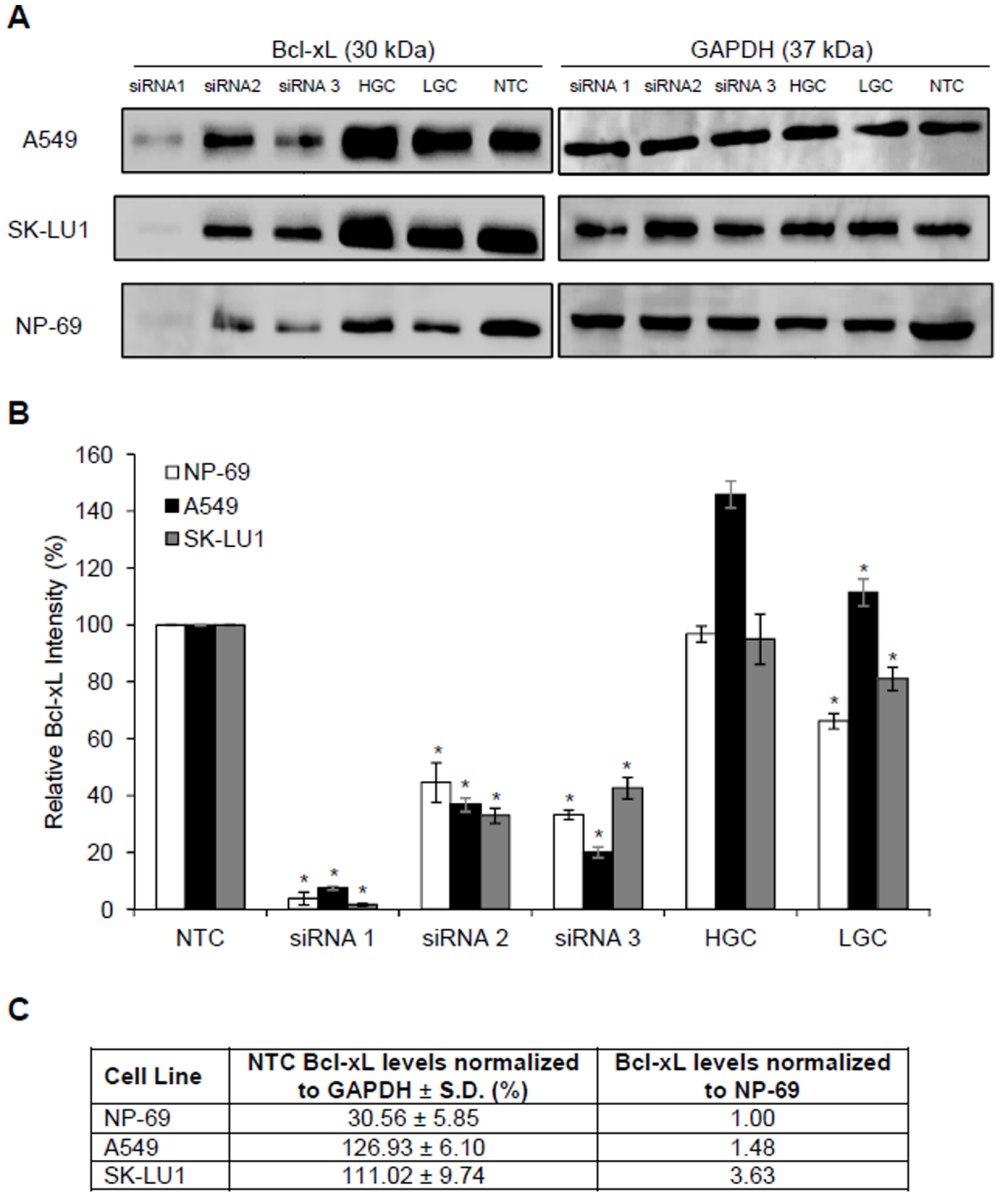
Bcl-xL protein levels were decreased in response to siRNA transfection in A549, SK-LU1 and NP-69 cells. (**A**) Western blot indicating a significant decrease in Bcl-xL protein levels in comparison to non-transfected cells 48 h post-transfection. GAPDH was used as a normalization control to ensure equal protein concentrations across samples. (**B**) Densitometry analysis of Western blot bands using the ImageJ Analyst software indicating a 92.47±0.72% reduction in Bcl-xL protein levels in A549 cells, a 98.33±0.50% reduction in SK-LU1 cells, and a 96.16±2.28% in NP-69 cells 48 h post-transfection with siRNA 1. (**C**) Table comparing Bcl-xL protein levels in non-transfected samples of NP-69, A549 and SK-LU1 cells normalized to GAPDH, as determined through densitometry analysis of Western blots. All experiments were carried out in triplicates, and presented as mean ± S.D. Statistically significant differences in Bcl-xL protein levels between non-transfected and transfected samples were indicated by (*) where *p*-value ≤0.05. HGC denotes cells transfected with high GC content scramble RNA negative control, while LGC denotes cells transfected with low GC content scramble RNA negative control. NTC denotes non-transfected cells.

**Figure 3:**
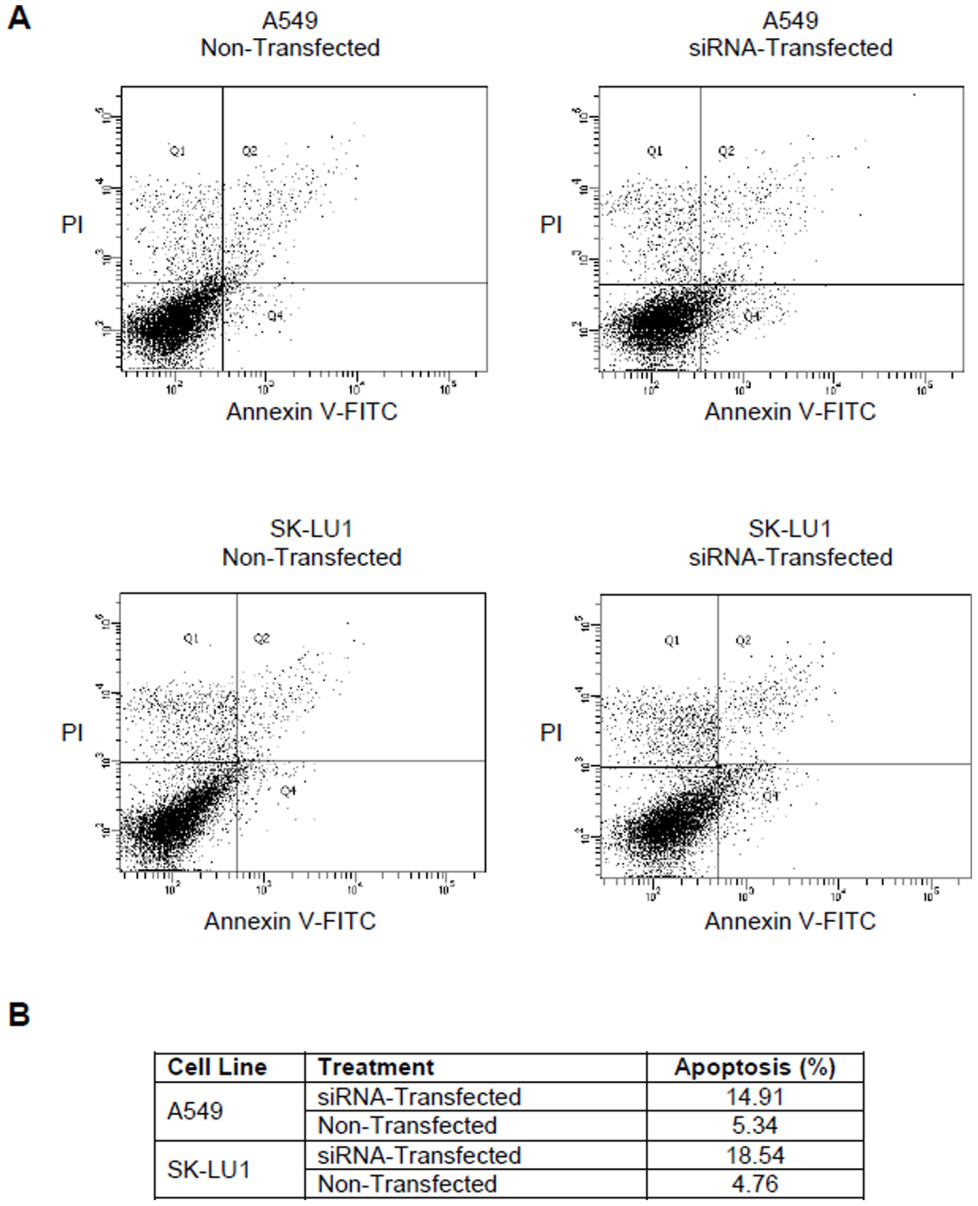
Detection of apoptosis using flow cytometry after annexin V-FITC/propidium iodide (PI) staining after 48 h. (**A**) siRNA-transfected and non-transfected A549 and SK-LU1 cells. Viable cells are in the lower left quadrant, early apoptotic cells are in the lower right quadrant, late apoptotic cells are in the upper right quadrant and non-viable necrotic cells are in the upper left quadrant. Dot plots are representative of 1.0×10^4^ cells from a single replicate. (**B**) Table comparing total apoptosis (%) as obtained from flow cytometer at 48 h post transfection.

The expression of *bcl-xL* in A549, SK-LU1 and NP-69 cells were silenced via transfection with Stealth RNAi™ siRNA Duplex Oligonucleotides. Visual monitoring of the uptake of BLOCK iT™ Alexa Fluor® Red Fluorescent Oligo using fluorescence microscopy showed a satisfactory transfection efficiency of ≥80.0% for all samples treated with the BLOCK-iT™ Alexa Fluor® Red Fluorescent Oligo (Fig. 1a and 1b). Complete transfection images are available in Figure S1. Silencing efficiency indicated a negative fold induction of 4.42±0.08 in A549 cells with a knockdown of 75.19±8.32% in *bcl-xL* expression when compared with non-transfected (NTC) A549 cells. SK-LU1 cells demonstrated a negative fold induction of 4.03±0.01 with a percentage knockdown of 75.16±0.92% in *bcl-xL* expression when compared with NTC (Fig. 1c and 1d). Correspondingly, Bcl-xL protein levels were decreased to 7.53±0.72% and 1.67±0.50% in A549 and SK-LU1 cells respectively, as determined by densitometry analysis of Western blot bands (Fig. 2). Densitometry analysis of the Western blot bands also revealed that native Bcl-xL protein levels in normal NP-69 cells were significantly lower than both lung adenocarcinoma cells lines, A549 and SK-LU1 upon normalization against GAPDH (Fig. 2C). As siRNA 1 had the greatest silencing efficiency amongst the three siRNAs, it was chosen for further downstream work. MTT cell viability assay was carried out and showed that a knockdown of *bcl-xL* gene expression also resulted in reduction in cell viability after 48 h post-transfection compared to NTC A549 and SK-LU1 cells (Table 1). Viability levels of NP-69 normal cells were found to be unaffected by the knockdown of *bcl-xL* gene expression. A comparison between NTC and mock transfected control (MTC) did not disclose any changes in viability, hence ruling out toxicity effects of the transfection reagent. A double fluorescence staining of annexin V-FITC conjugate and propidium iodide was performed on *Bcl-xL* silenced cells and non-silenced cells, and analyzed using a flow cytometer to determine if cells were dying through apoptosis. After silencing of *bcl-xL* in both A549 and SK-LU1 cells, the population of cells indicated a shift from viable cells to early and late stage apoptosis with an increase of apoptosis by 9.57% and 13.78% respectively (Fig. 3).

**Table 1:** Total viability levels (%) from MTT assays indicating increased cytotoxicity levels upon siRNA-based *bcl-xL* silencing.

Cell Lines	Treatment	Time (h)	Viability (%) ± S.D.	*p-*value
NP-69	NTC†	12	115.12±3.90	n/a
		24	111.21±12.13	n/a
		48	109.54±4.30	n/a
	MTC†	12	95.73±6.84	0.065
		24	98.84±12.06	0.494
		48	93.46±13.19	0.248
	siRNA	12	101.77±7.70	0.094
		24	93.61±1.83	0.130
		48	92.69±3.08	0.057
A549	NTC†	12	104.17±9.19	n/a
		24	109.28±9.67	n/a
		48	102.66±6.85	n/a
	MTC†	12	92.77±12.29	0.328
		24	98.12±1.70	0.224
		48	97.79±2.22	0.430
	siRNA	12	83.85±1.88	0.036
		24	81.26±2.84	0.052
		48	58.69±6.15	0.003
SK-LU1	NTC†	12	104.57±5.61	n/a
		24	103.61±2.12	n/a
		48	103.29±3.69	n/a
	MTC†	12	97.95±3.03	0.306
		24	97.95±7.56	0.289
		48	95.58±11.97	0.250
	siRNA	12	89.29±8.06	0.009
		24	72.80±1.15	0.003
		48	58.22±0.98	0.002

All experiments were carried out with three independent biological replicates and presented as mean ± S.D. Results with *p*≤0.05 in comparison to NTC were considered statistically significant.

† NTC denotes non-transfected control; MTC denotes mock-transfected control.

### 3.2 Ten miRNAs Found to be Significantly Dysregulated in *bcl-xL* Silenced A549 Cells

**Figure 4:**
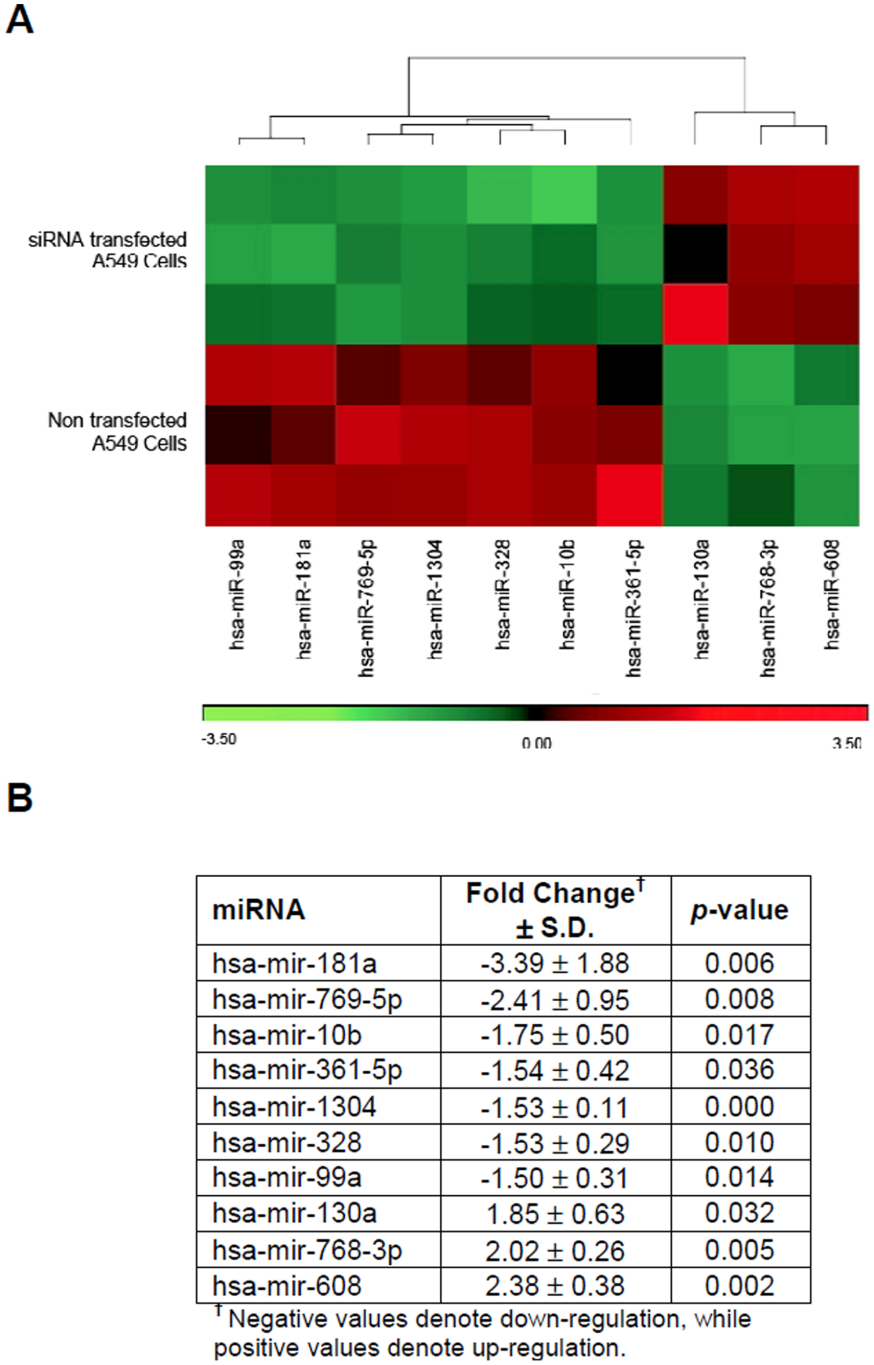
MiRNA profile in siRNA-silenced A549 cells. (**A**) Hierarchical clustering heat map of 10 differentially expressed miRNAs in siRNA-transfected A549 cells versus non-transfected A549 cells. Up-regulated miRNAs are represented in red, while down-regulated miRNAs are represented in green. (**B**) List of differentially expressed miRNAs filtered with ≥1.5-fold change expression and *p*≤0.05 using the Partek® Genomics™ Suite (PGS) software. All experiments were carried out with three independent biological replicates and presented as mean ± S.D.

**Figure 5:**
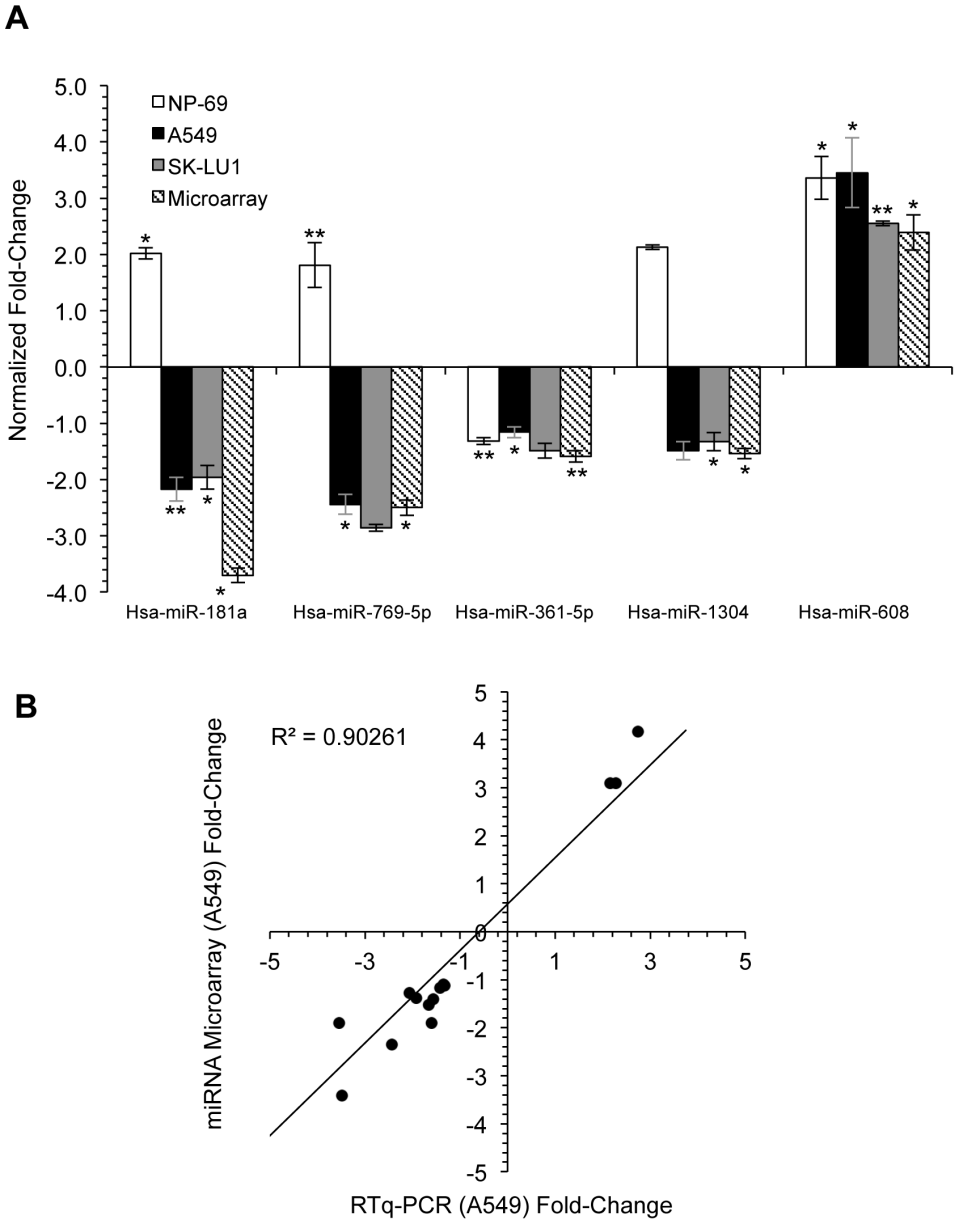
Correlation between miRNA microarray data and RT-qPCR data. (**A**) RT-qPCR of the five miRNAs (hsa-miR-181a, hsa-miR-769-5p, hsa-miR-361-5p, hsa-miR-1304, and hsa-miR-608) validated against microarray results, and presented as normalized fold-change values. All experiments were carried out with three independent biological replicates and presented as mean ± S.D. Statistically significant differences in miRNA fold-change between non-transfected and transfected samples were indicated by *p-value ≤0.01 and **p-value ≤0.05. (**B**) Pearson correlation scatter plot between two variables, A549 miRNA microarray fold-change and A549 RT-qPCR fold-change of five validated miRNAs (hsa-miR-181a, hsa-miR-769-5p, hsa-miR-361-5p, hsa-miR-1304, and hsa-miR-608) from three independent biological replicates, produced a correlation coefficient value of *r* = 0.950 with an *r^2^* = 0.903, indicating a strong positive correlation between both sets of data.

To determine miRNA expression changes that occur in response to *bcl-xL* silencing, a global miRNA expression profile was established using miRNA microarray, which compared total RNA, extracted from siRNA-transfected and non-transfected A549 cells (Fig. 4a). We identified 10 miRNAs that were significantly differentially expressed of which, 7 miRNAs were down-regulated while 3 were up-regulated. Among down-regulated miRNAs, hsa-mir-181a was found to display the highest reduction in expression with a fold change of −3.39±1.88, while hsa-mir-608 had the highest increase in expression with a fold change of 2.38±0.38 (Fig. 4b). Five representative differentially expressed miRNAs (hsa-miR-181a, hsa-miR-769-5p, hsa-miR-361-5p, hsa-miR-1304, and hsa-miR-608) were selected to undergo RT-qPCR validation based on highest fold-change as well as putative targets as identified by the TargetScan web tool (Fig. 5a). Data validation indicated a highly positive correlation between RT-qPCR data and miRNA microarray with a correlation coefficient value, *r* of 0.950 and *r* ^2^ of 0.903 (Fig. 5b). RT-qPCR indicated that miR-181a, miR-769-5p, and miR-361-5p were all down-regulated in siRNA-transfected A549 and SK-LU1 cells when compared to non-transfected cells, while miR-608 was up-regulated. However, RT-qPCR performed on NP-69 normal cells indicated opposing results in relation to miR-181a, miR-769-5p and miR-1304 expression, with these miRNAs being up-regulated. This suggests that dysresgulation of these miRNAs may be cancer cell specific. This prompted us to further identify the specific cancer-related gene targets, which were regulated by these miRNAs and were responsible for the increased levels of cytotoxicity in *bcl-xL* silenced cells.

### 3.3 Gene Targets of Selected miRNAs Dysregulated in *bcl-xL* Silenced A549 Cells

Bioinformatics analysis using TargetScan Human v5.2 on the five selected dysregulated miRNAs which predicted gene targets with total context scores of <0 were selected for gene-annotation enrichment analysis using DAVID v6.7. These miRNAs were found to be associated with various signaling pathways, primarily the phosphatidylinositol 3-kinase/protein kinase B (PI3K/AKT), wingless-type MMTV integration site family (WNT), transforming growth factor (TGF-β), mitogen activated protein kinase (MAPK) and the intrinsic pathway. A full list of miRNA predicted gene targets can be found under Table S1.

### 3.4 Up-regulation of hsa-miR-608 Expression Increases Cell Death in A549 and SK-LU1 Cells

**Figure 6:**
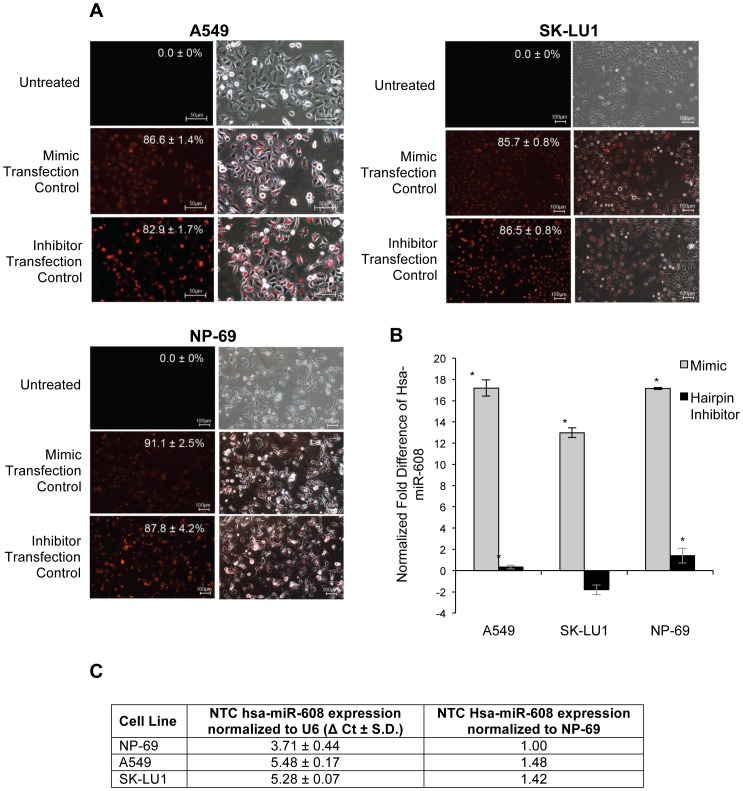
Transfection and quantification of hsa-mir-608 mimics and hairpin inhibitors. (**A**) Fluorescent and merged images of A549, SK-LU1 and NP-69 cells transfected with miRIDIAN microRNA Mimic/Hairpin Inhibitor Transfection Control with Dy547. Percentage of mean transfection efficiency is indicated, and all images shown are a representative of triplicates independent experiments. (**B**) RT-qPCR of hsa-miR-608 presented as normalized fold difference in mimic/hairpin inhibitor-transfected A549, SK-LU1 and NP-69 cells, in comparison to cells transfected with scrambled negative controls. (**C**) Table comparing the fold-difference of hsa-miR-608 in mimic/hairpin inhibitor-transfected A549, SK-LU1 and NP-69 cells in comparison to scrambled negative control cells. All experiments were carried out with three independent biological replicates and presented as mean ± S.D. Statistically significant differences in relative expression between scrambled negative controls and transfected samples were indicated by (*) where *p*-value ≤0.05. NTC denotes non-transfected cells. MTC indicates mock transfected cells with transfection reagent only.

**Figure 7:**
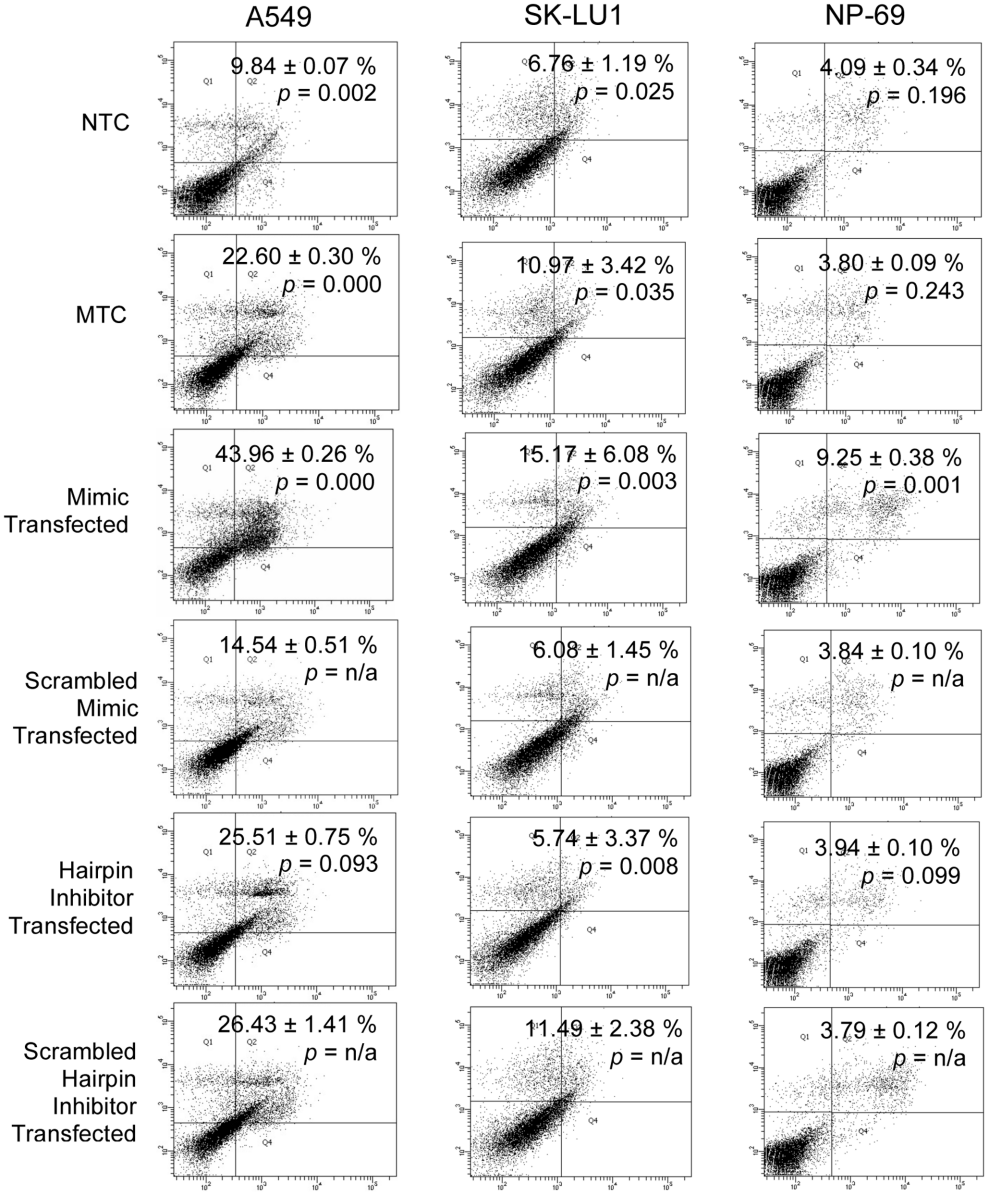
Detection of apoptosis after annexin V-FITC/PI staining after transfection with mimics or hairpin inhibitors. Results indicated that up-regulation of hsa-miR-608 expression potentiates apoptosis-mediated cell death after 72 h in A549 and SK-LU1 cells, while NP-69 cells were minimally affected by hsa-miR-608 overexpression. Viable cells are in the lower left quadrant, early apoptotic cells are in the lower right quadrant, late apoptotic cells are in the upper right quadrant and non-viable necrotic cells are in the upper left quadrant. Dot plots are representative of 1.0×10^4^ cells from a single replicate with percentage of apoptosis indicated. MTC denotes cells transfected with transfection reagent only, while NTC denotes non-transfected cells. Scrambled miRNA mimics and anti-sense hairpin inhibitor sequences were used as negative controls.

Hsa-miR-608 was chosen from the list of dysregulated miRNAs and its expression was up-regulated to observe its effects on cell death in A549, SK-LU1 and NP-69 cells. Monitoring of miRIDIAN microRNA Mimic and Inhibitor Transfection Control with Dy547 uptake using fluorescence microscopy showed a transfection efficiency of ≥80% across all samples (Fig. 6a). Quantification of hsa-miR-608 overexpression by RT-qPCR indicated a positive fold difference of 17.19±0.76, 12.99±0.46, and 17.15±0.09 in A549, SK-LU1 and NP-69 transfected cells respectively, when compared with cells transfected with scrambled negative controls (Fig. 6b). Relative quantitation of miRNA and complete fold difference results are available in Table S2. Results also revealed that the relative quantity of hsa-miR-608 were lower in NTC samples of normal NP-69 cells in comparison to NTC samples of both lung adenocarcinoma cells, A549 and SK-LU1 (Fig. 6c). An increase in hsa-miR-608 consequently resulted in an increase in apoptotic population amongst A549 (43.85±0.26%) and SK-LU1 (15.17±1.27) cells after 72 h of transfection, while only 9.89±0.07% and 6.76±1.19% total apoptosis was observed in NTC cells of A549 and SK-LU1 cells respectively (Fig. 7). On the other hand, knockdown of hsa-miR-608 with hairpin anti-sense inhibitor sequences resulted in lower apoptotic population in comparison to mimic transfected cells, with 25.51±0.75% in A549 cells, 5.74±3.37% in SK-LU1 and 3.94±0.10% in NP-69 (Fig. 7).

### 3.5 Transfection with hsa-miR-608 Anti-sense Inhibitors Blocks si*Bcl-xL* Induced Cell Death

**Figure 8:**
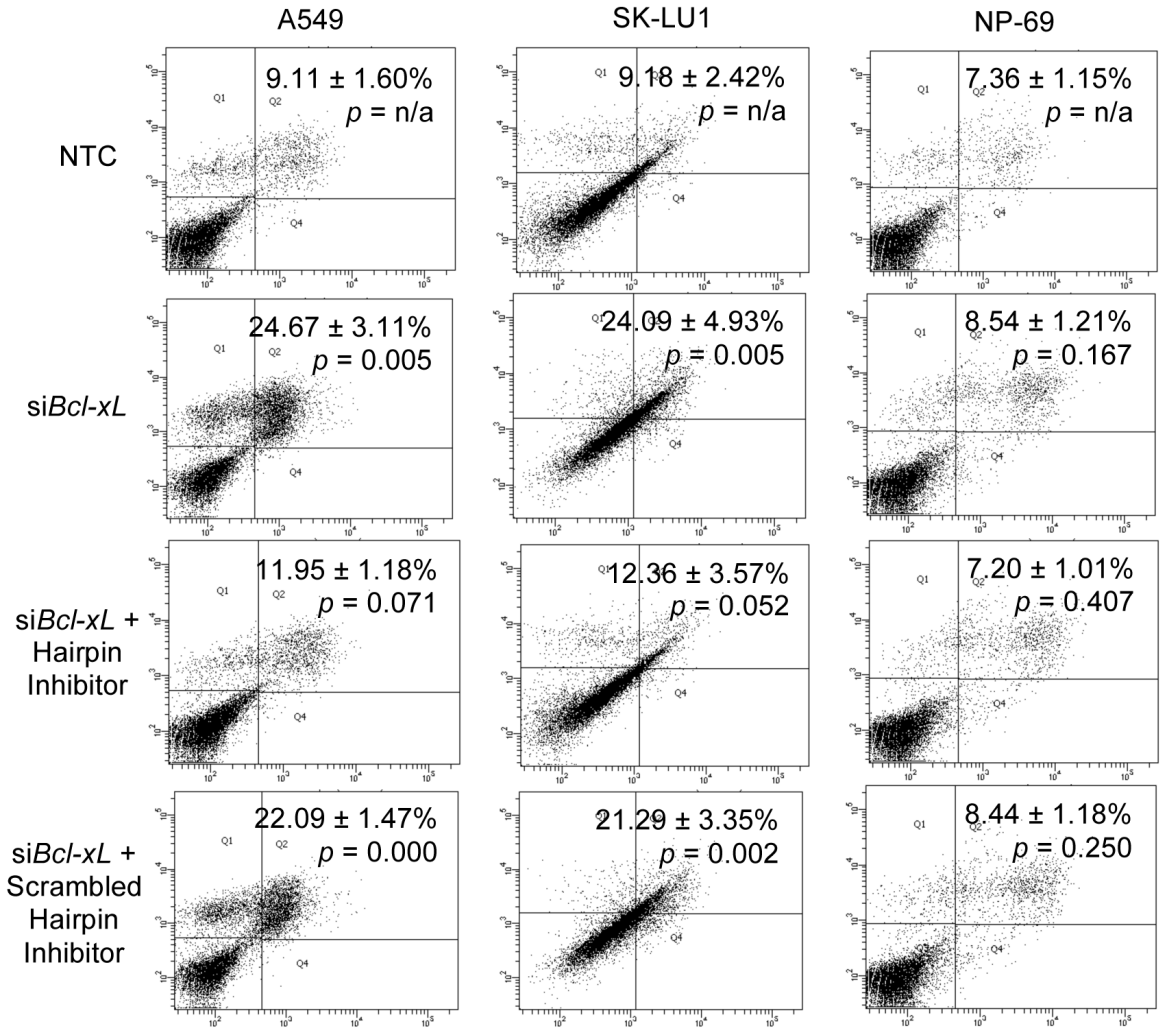
Annexin V-FITC/PI staining indicating that transfection of hsa-miR-608 hairpin inhibitors prevents si*Bcl-xL* induced cell death. Dot plots are representative of 1.0×10^4^cells from a single replicate with percentage of apoptosis from bottom right and top right quadrants indicated. NTC denotes non-transfected cells while si*Bcl-xL* indicates cells transfected with Bcl-xL-silencing siRNAs. Scrambled anti-sense hairpin inhibitor sequences were used as negative controls.

To determine the relationship between *bcl-xL*, hsa-miR-608 and cell death, a combination study was carried out whereby cells were first transfected with si*Bcl-xL*, followed by transfection with hsa-miR-608 antisense inhibitors. It was found that apoptotic populations of *bcl-xL* silenced A549 and SK-LU1 cells were significantly decreased following hairpin inhibitors transfection indicating that antagomiRs of hsa-miR-608 was able to block si*Bcl-xL* induced cell death, thus suggesting that hsa-miR-608 plays an important role in cell death processes (Fig. 8). A comparison between si*Bcl-xL* transfected cells and si*Bcl-xL* with hairpin anti-sense inhibitors did not disclose any significant changes in apoptotic population of NP-69 cells, hence indicating that the apoptotic population of NP-69 was unaffected by the silencing of *bcl-xL*.

## Discussion

Previous studies have shown that *bcl-xL* anti-sense treatment evokes a strong apoptotic response in lung adenocarcinoma cells that lack the significant expression of Bcl-2 [22]. However small-cell lung cancer (SCLC) cells did not undergo apoptosis following the same *bcl-xL* down-regulation, most probably due to high levels of Bcl-2 which counters the lacking presence of Bcl-xL, thereby providing apoptotic protection [22]. These findings suggest that Bcl-xL is a more critical apoptosis repressor protein in NSCLCs such as lung adenocarcinoma cells than in SCLCs [22], [23]. In this study, siRNA-based silencing of the *bcl-xL* gene led to a decrease in cell proliferation of A549 and SK-LU1 cells but not in NP-69 cells. This corresponded with previous Bcl-xL antisense treatments, suggesting Bcl-xL as the critical apoptosis repressor protein in lung adenocarcinoma cell lines but not in untransformed cell types. Despite observing the presence of apoptosis-mediated cell death via annexin V-FITC/PI assays which associates cell death via phosphatidylserine externalization and membrane integrity, the specific mechanism by which cell death occurs cannot be attributed solely towards *bcl-xL* inhibition alone. Possible rippling effects of miRNA expression alterations toward gene targets associated with other cell death-inducing factors such as Fas-mediation, TNF-induction, and other caspase independent pathways cannot be ruled out. To date, no studies have been conducted to identify miRNAs that are regulated or affected by the expression of *bcl-xL.* As miRNAs are critical epigenetic apoptosis regulators in tumorigenesis, and cancer cells often have perturbed miRNA profiles that regulate cell survival, it is of interest to determine the role miRNAs play in the apoptotic properties of Bcl-xL silenced human lung adenocarcinoma cells.

Previously, studies have been carried out which have implicated dysregulated miRNAs from this study in a wide variety of cancers. For example, miR-181a is up-regulated in thyroid papillary carcinomas [24], but down-regulated in glioblastomas [25]. In a separate study, it was found that miR-181a in human glioma cells function as a tumor suppressor with the ability to induce apoptosis and inhibit division [25]. In NSCLC cells, expression of miR-181a has been found to be down-regulated compared to normal lung cells, and was negatively correlated with patient survival time [26]. Furthermore, miR-181a acts as a chemosensitizer enhancing the lethality of various chemotherapeutic agents such as cisplatin, carboplatin, and oxaliplatin in several NSCLCs by stimulating Bax oligomerization and activation of pro-apoptotic caspases [27]. As for miR-328, its expression was found to be inversely correlated to ATP-binding cassette subfamily G(ABCG2) levels in human breast cancer cells [28]. Overexpression of ABCG2 represents an important mechanism for multidrug resistance where it significantly increases drug sensitivity in cancer cells [28]. While many other studies have been carried out linking the 10 dysregulated miRNAs in this study with various cancers, their association with lung adenocarcinoma cell death is still lacking.

In this study we have shown that *bcl-xL* levels play a role in the expression of miRNAs in lung adenocarcinoma cells. Bioinformatic analyses predicted that the five significantly dysregulated miRNAs (hsa-miR-181a, hsa-miR-769-5p, hsa-miR-361-5p, hsa-miR-1304, and hsa-miR-608) were linked to various apoptotic signaling pathways, including the PI3K/AKT, WNT, TGF-β, MAPK/ERK and the intrinsic pathway, and all were implicated as those directly affected by Bcl-xL levels. Both Akt and TGF-β plays a significant role in the activation of a number of processes critical for tumorigenesis including inhibition of apoptosis, aberrant cell proliferation, promotion of angiogenesis and tumor cell invasiveness [29]–[31]. In the intrinsic pathway, the breakpoint cluster region/Abelson (BCR/Abl) fusion protein, STAT5 and Bcl-xL were all predicted targets of miRNAs. BCR/Abl is an oncogene that is able to activate STAT5, which has been found to be increased in lung cancer cell lines, and was speculated to control the process of apoptosis through the up-regulation of the anti-apoptotic protein Bcl-xL [32], [33]. Members of the MAPK pathway were also predicted to be targets of dysregulated miRNAs, specifically the extracellular signal-regulated kinase (ERK) pathway. Activation of K-Ras, which was a predicted target, leads to stimulation of various pathways, primarily the Raf-MEK-ERK pathway and the PI3K pathway, that consequently results in tumor cell growth and proliferation, apoptosis, metastasis, invasion and angiogenesis [34], [35]. A combined dysregulation of genes from these signaling pathways may account for the increase in cell death observed in A549 and SK-LU1 following silencing of *Bcl-xL*, however further studies must be carried out to determine the specific functions of these miRNAs.

While hsa-miR-608 was significantly up-regulated in NP-69 cells following transfection with si*Bcl-xL*, it was found that opposing results were observed in NP-69 cells in regards to the expression of miR-181a, miR-769-5p and miR-1304, in comparison to A549 and SK-LU1 cells (Fig. 5a). Subsequently, reduction in cell viability and increase in cell death was only observed in A549 and SK-LU1 while NP-69 cells were minimally affected. As all these miRNAs are hypothesized to also play a role in the cell death process, we postulated that these miRNAs work in synergy to achieve this goal. Thus it can be expected that the attenuation of target gene expression, and in turn cellular processes by individual miRNAs can be quite modest [36]–[38].

MiRNAs have the ability to work together to augment the functions of individual gene targets; conversely they are able to target multiple genes that are co-expressed or act in a common pathway. For example in a study conducted by Krek and colleagues, it was discovered that miR-375, miR-124 and let-7b share a common gene target, *Mtpn* [39]. It was found that combining miR-124 and let-7b led to a decrease in expression of *Mtpn*, similar to that caused by miR-175 alone. However a combined addition of miR-124 and let-7b together with miR-375 lead to a substantially greater target inhibition than any effects observed by any other combinations [39]. In another study in 2008, Ivanovska and Cleary identified three miRNAs (miR-34a, miR-16 and miR-106b) that participate in regulating networks of genes that participate in a common cellular process, but through distinct molecular mechanisms. They investigated the possibility for synergies among the miRNAs and discovered that by combining miR-34a and miR-16 lead to a G_1_ block that was greater than that exhibited by each miRNA alone. Furthermore, when miR-106b was combined with miR-34a, miR-16, or both, an intermediate phenotype was observed, which reflected the contributions from each miRNA [40].

Furthermore, our results indicated lower basal levels of Bcl-xL protein in non-transfected NP-69 cells (Fig. 2c), which will correspondingly lead to lower levels of hsa-miR-608 levels in non-transfected NP-69 cells in comparison to the non-transfected A549 and SK-LU1 cells (Fig. 6c). Therefore, even with comparable fold increase in hsa-miR-608 following siRNA silencing of Bcl-xL, the relative quantity of hsa-miR-608 in NP-69 cells would still be lower than that observed in A549 and SK-LU1. As hsa-miR-608 is suggested to play a role in cell death, a lower hsa-miR-608 level in NP-69 may account for the lack of changes in cell viability and apoptotic population that was observed in NP-69 transfected cells (Table 1 & Fig. 8).

## Conclusions

This study describes the successful determination of miRNAs dysregulated in response to *bcl-xL* silencing in lung adenocarcinoma NSCLC cells, with the aim to elucidate the related hypothetical pathways involved in the induction of cell death. Ten significantly dysregulated miRNAs were linked to several apoptotic signaling pathways including the PI3K/AKT, WNT, TGF-β, MAPK/ERK and the intrinsic pathway, and all were implicated as those directly affected by Bcl-xL levels. With further studies carried out to determine the specific functions of these miRNAs, our study has provided a platform for anti-sense treatment whereby miRNA expression can be exploited to increase the apoptotic properties in lung adenocarcinoma cells as demonstrated by our overexpression and knockdown studies on hsa-mir-608.

## Supporting Information

Figure S1 **Silencing of** ***bcl-xL*** **using siRNA-based transfection.** Fluorescent image and merged image of A549 and SK-LU1 cells transfected with BLOCK-iT™ Alexa Fluor® Red Fluorescent Oligo. Percentage of mean transfection efficiency is indicated, and all images shown are a representative of triplicates independent experiments. HGC denotes cells transfected with high GC content scramble RNA negative control. LGC denotes cells transfected with low GC content scramble RNA negative control.
(TIF)

Table S1 Bioinformatics analysis of miRNA targets. List of miRNA gene targets as obtained using TargetScan 5.2 software with a context score threshold of ≤0.
(DOCX)

Table S2 Determination of hsa-miR-608 mimics and hairpin inhibitors fold change and relative quantity. **(A)** Table comparing the relative quantity of hsa-miR-608 in mimic/hairpin inhibitor-transfected A549, SK-LU1 and NP-69 cells in comparison to levels in scrambled negative control cells. **(B)** Table comparing the fold difference of hsa-miR-608 in mimic/hairpin inhibitor-transfected A549, SK-LU1 and NP-69 cells in comparison to scrambled negative control cells. **(C)** Table comparing the fold difference of hsa-miR-608 in MTC and scrambled negative control transfected A549, SK-LU1 and NP-69 cells in comparison to NTC cells. All experiments were carried out with three independent biological replicates and presented as mean ± S.D. Statistically significant differences in relative expression between scrambled negative controls and transfected samples were indicated by (*) with a *p*-value ≤0.05. NTC denotes non-transfected cells. MTC indicates cells transfected with transfection reagent only.
(DOCX)
