# Supplementary figures and images for: Bcl-xL Silencing Induces Alterations in hsa-miR-608 Expression and Subsequent Cell Death in A549 and SK-LU1 Human Lung Adenocarcinoma Cells
Source: PLoS One. 2013 Dec 10;8(12):e81735. doi: 10.1371/journal.pone.0081735 (PMC3858247; doi:10.1371/journal.pone.0081735)

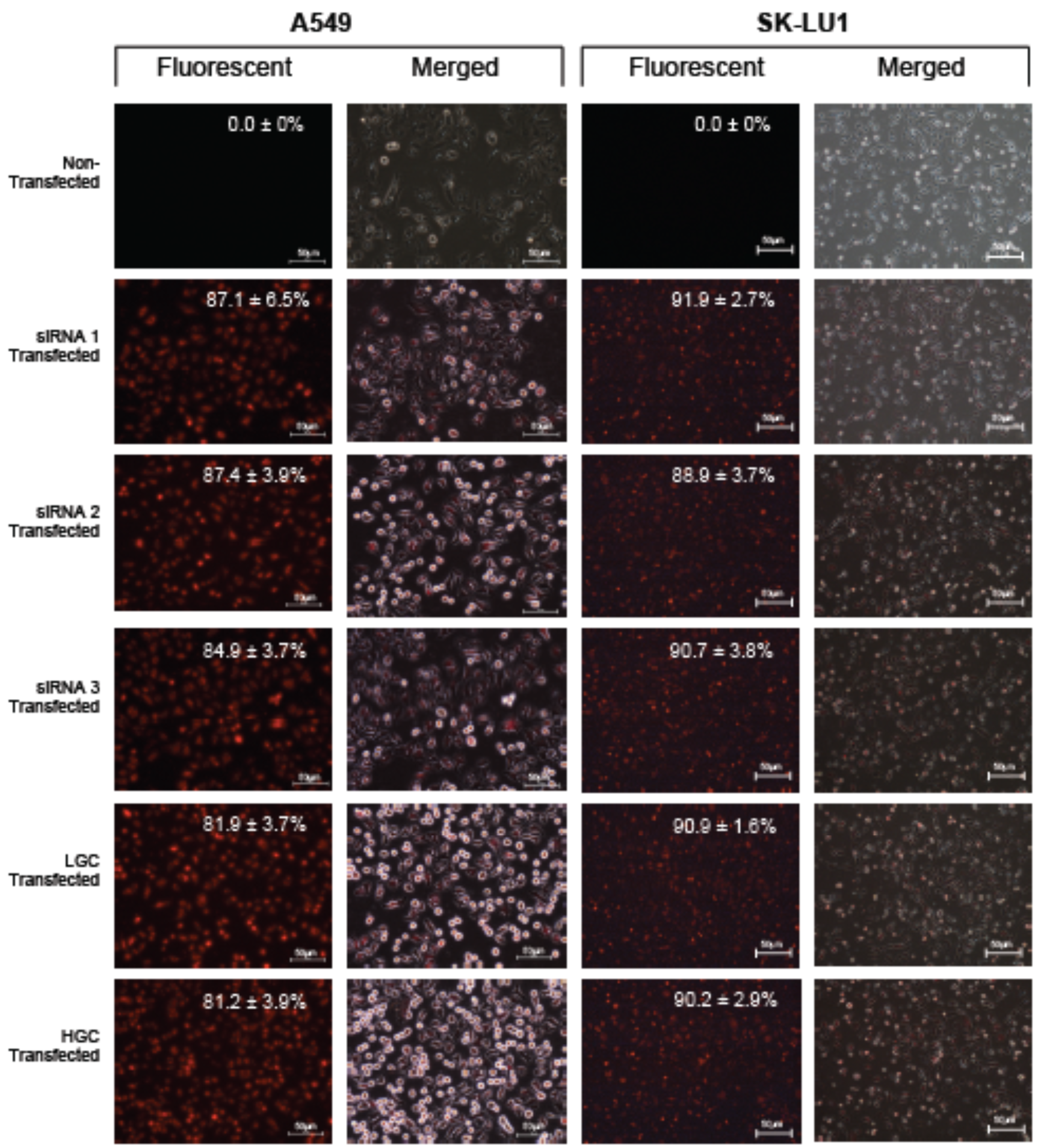

Supplement: Figure S1 — Silencing of bcl-xL using siRNA-based transfection. Fluorescent image and merged image of A549 and SK-LU1 cells transfected with BLOCK-iT™ Alexa Fluor® Red Fluorescent Oligo. Percentage of mean transfection efficiency is indicated, and all images shown are a representative of triplicates independent experiments. HGC denotes cells transfected with high GC content scramble RNA negative control. LGC denotes cells transfected with low GC content scramble RNA negative control. (TIF) [file pone.0081735.s001.tif]
