# Supplementary material for: Bcl-xL Silencing Induces Alterations in hsa-miR-608 Expression and Subsequent Cell Death in A549 and SK-LU1 Human Lung Adenocarcinoma Cells
Source: PLoS One. 2013 Dec 10;8(12):e81735. doi: 10.1371/journal.pone.0081735 (PMC3858247; doi:10.1371/journal.pone.0081735)
Supplement: Table S1 — Bioinformatics analysis of miRNA targets. List of miRNA gene targets as obtained using TargetScan 5.2 software with a context score threshold of ≤0. (DOCX) [file pone.0081735.s002.docx]

| Gene Symbol | Gene Description | TargetScan Total Context Score | | | | |
| --- | --- | --- | --- | --- | --- | --- |
|  |  | **hsa-miR-181a** | **hsa-miR-769-5p** | **hsa-miR-361-5p** | **hsa-miR-1304** | **hsa-miR-608** |
| AKT2 | v-akt murine thymoma viral oncogene homolog 2 | -0.21 | -0.02 | N/A | N/A | -0.17 |
| APC2 | adenomatosis polyposis coli 2 | N/A | 0.05 | N/A | -0.03 | -0.33 |
| BCL2 | B-cell CLL/lymphoma 2 | N/A | N/A | N/A | -0.15 | N/A |
| BCR | breakpoint cluster region | -0.05 | -0.05 | N/A | -0.14 | -0.16 |
| CASP3 | caspase 3, apoptosis-related cysteine peptidase | N/A | N/A | N/A | -0.12 | N/A |
| CASP9 | caspase 9, apoptosis-related cysteine peptidase | N/A | N/A | N/A | -0.21 | -0.12 |
| CCND1 | cyclin D1 | N/A | N/A | -0.12 | N/A | -0.31 |
| CDK6 | cyclin-dependent kinase 6 | -0.04 | N/A | N/A | -0.11 | -0.16 |
| CYCS | cytochrome c, somatic | N/A | -0.07 | N/A | -0.16 | N/A |
| E2F2 | E2F transcription factor 2 | -0.03 | 0.06 | N/A | N/A | -0.13 |
| EGFR | epidermal growth factor receptor | N/A | N/A | N/A | N/A | -0.56 |
| FADD | Fas (TNFRSF6)-associated via death domain | N/A | N/A | -0.44 | N/A | N/A |
| FAS | Fas (TNF receptor superfamily, member 6) | -0.44 | N/A | -0.32 | -0.08 | -0.19 |
| FASLG | Fas ligand (TNF superfamily, member 6) | -0.16 | N/A | N/A | N/A | N/A |
| FLT3 | fms-related tyrosine kinase 3 | N/A | N/A | -0.14 | N/A | N/A |
| FOS | v-fos FBJ murine osteosarcoma viral oncogene homolog | -0.16 | N/A | N/A | -0.11 | N/A |
| FOXO1 | forkhead box O1 | N/A | N/A | -0.05 | N/A | N/A |
| FZD1 | frizzled homolog 1 | N/A | N/A | N/A | -0.15 | N/A |
| HGF | hepatocyte growth factor (hepapoietin A; scatter factor) | N/A | N/A | N/A | -0.19 | N/A |
| LEF1 | lymphoid enhancer-binding factor 1 | N/A | N/A | N/A | N/A | -0.08 |
| MAP2K1 | mitogen-activated protein kinase kinase 1 | -0.42 | N/A | N/A | N/A | N/A |
| MAP2K2 | mitogen-activated protein kinase kinase 2 | N/A | -0.03 | N/A | N/A | N/A |
| MDM2 | Mdm2 p53 binding protein homolog | N/A | N/A | N/A | -0.29 | N/A |
| MET | met proto-oncogene (hepatocyte growth factor receptor) | -0.19 | N/A | -0.61 | N/A | -0.1 |
| MYC | v-myc myelocytomatosis viral oncogene homolog | N/A | N/A | N/A | -0.46 | N/A |
| NFKB2 | nuclear factor of kappa light polypeptide gene enhancer in B-cells 2 (p49/p100) | N/A | N/A | N/A | N/A | -0.35 |
| PDGFB | platelet-derived growth factor beta polypeptide | N/A | -0.1 | N/A | N/A | -0.18 |
| PDGFRA | platelet-derived growth factor receptor, alpha polypeptide | N/A | N/A | -0.11 | N/A | N/A |
| PIK3R1 | phosphoinositide-3-kinase, regulatory subunit 1 (alpha) | -0.08 | -0.04 | -0.27 | -0.05 | -0.22 |
| PPARD | peroxisome proliferator-activated receptor delta | N/A | 0.08 | N/A | N/A | -0.15 |
| PRKCA | protein kinase C, alpha | -0.04 | -0.01 | N/A | -0.18 | -0.17 |
| SMAD4 | SMAD family member 4 | N/A | N/A | -0.14 | N/A | N/A |
| SOS2 | son of sevenless homolog 2 | N/A | N/A | N/A | N/A | -0.17 |
| TCF7 | transcription factor 7 (T-cell specific, HMG-box) | N/A | -0.06 | N/A | -0.18 | N/A |
| TGFA | transforming growth factor, alpha | -0.01 | N/A | -0.01 | -0.18 | N/A |
| TGFBR1 | transforming growth factor, beta receptor 1 | N/A | -0.15 | -0.21 | N/A | N/A |
| TP53 | tumor protein p53 | N/A | -0.15 | N/A | N/A | -0.28 |
| TRAF2 | TNF receptor-associated factor 2 | N/A | -0.03 | N/A | N/A | N/A |
| TRAF3 | TNF receptor-associated factor 3 | N/A | N/A | -0.3 | N/A | N/A |
| TRAF5 | TNF receptor-associated factor 5 | -0.17 | N/A | -0.26 | N/A | N/A |
| TRAF6 | TNF receptor-associated factor 6 | N/A | N/A | N/A | N/A | -0.16 |
| WNT1 | wingless-type MMTV integration site family, member 1 | N/A | N/A | N/A | 0.00 | N/A |
| XIAP | X-linked inhibitor of apoptosis | -0.24 | N/A | N/A | N/A | N/A |
