# Supplementary material for: Bcl-xL Silencing Induces Alterations in hsa-miR-608 Expression and Subsequent Cell Death in A549 and SK-LU1 Human Lung Adenocarcinoma Cells
Source: PLoS One. 2013 Dec 10;8(12):e81735. doi: 10.1371/journal.pone.0081735 (PMC3858247; doi:10.1371/journal.pone.0081735)
Supplement: Table S2 — Determination of hsa-miR-608 mimics and hairpin inhibitors fold change and relative quantity. (A) Table comparing the relative quantity of hsa-miR-608 in mimic/hairpin inhibitor-transfected A549, SK-LU1 and NP-69 cells in comparison to levels in scrambled negative control cells. (B) Table comparing the fold difference of hsa-miR-608 in mimic/hairpin inhibitor-transfected A549, SK-LU1 and NP-69 cells in comparison to scrambled negative control cells. (C) Table comparing the fold difference of hsa-miR-608 in MTC and scrambled negative control transfected A549, SK-LU1 and NP-69 cells in comparison to NTC cells. All experiments were carried out with three independent biological replicates and presented as mean ± S.D. Statistically significant differences in relative expression between scrambled negative controls and transfected samples were indicated by (*) with a p-value ≤0.05. NTC denotes non-transfected cells. MTC indicates cells transfected with transfection reagent only. (DOCX) [file pone.0081735.s003.docx]

**A**

| **Treatment** | **A549** | | **SK-LU1** | | **NP-69** | |
| --- | --- | --- | --- | --- | --- | --- |
|  | **Relative Quantity^†^ ± S.D** | ***p-*value** | **Relative Quantity^†^ ± S.D** | ***p-*value** | **Relative Quantity^†^ ± S.D** | ***p-*value** |
| NTC | 1.20 ± 0.03 | 0.0040 | -7.14 ± 0.09 | 0.002 | -5.06 ± 0.01 | 0.0000 |
| MTC | -1.25 ± 0.15 | 0.0751 | -14.29 ± 0.02 | 0.0001 | 1.22 ± 0.20 | 0.1015 |
| Mimic | 164589.71 ± 90879.57 | 0.0003 | 8401.4 ± 2457.27 | 0.0002 | 145,271.60 ± 8749.68 | 0.0000 |
| Scrambled Mimic | 1.00 ± 0.00 | n/a | 1.00 ± 0.00 | n/a | 1.00 ± 0.00 | n/a |
| Hairpin Inhibitor | 1.27 ± 0.15 | 0.0393 | -1.42 ± 0.22 | 0.0872 | 2.85 ± 1.23 | 0.0364 |
| Scrambled Hairpin Inhibitor | 1.00 ± 0.00 | n/a | 1.00 ± 0.00 | n/a | 1.00 ± 0.00 | n/a |

**^†^** Negative values denote decrease in relative quantity, while positive values denote increase in relative quantity

**B**

| **Treatment** | **A549** | | **SK-LU1** | | **NP-69** | |
| --- | --- | --- | --- | --- | --- | --- |
|  | **Fold Difference^††^ ± S.D** | ***p-*value** | **Fold Difference^††^ ± S.D** | ***p-*value** | **Fold Difference^††^ ± S.D** | ***p-*value** |
| NTC | 0.26 ± 0.04 | 0.0034 | -3.10 ± 1.14 | 0.0211 | -2.34 ± 0.04 | 0.0002 |
| MTC | -0.34 ± 0.26 | 0.0783 | -3.78 ± 0.44 | 0.0022 | 0.27 ± 0.25 | 0.0993 |
| Mimic | 17.19 ± 0.76 | 0.0003 | 12.99 ± 0.46 | 0.0002 | 17.15 ± 0.09 | 0.0000 |
| Scrambled Mimic | 0.00 ± 0.00 | n/a | 0.00 ± 0.00 | n/a | 0.00 ± 0.00 | n/a |
| Hairpin Inhibitor | 0.33 ± 0.17 | 0.0393 | -1.81 ± 0.460 | 0.0872 | 1.41 ± 0.70 | 0.0364 |
| Scrambled Hairpin Inhibitor | 0.00 ± 0.00 | n/a | 0.00 ± 0.00 | n/a | 0.00 ± 0.00 | n/a |

**^††^** Negative values denote down-regulation, while positive values denote up-regulation

**C**

| **Treatment** | **A549** | | **SK-LU1** | | **NP-69** | |
| --- | --- | --- | --- | --- | --- | --- |
|  | **Fold Difference^††^ ± S.D** | ***p-*value** | **Fold Difference^††^ ± S.D** | ***p-*value** | **Fold Difference^††^ ± S.D** | ***p-*value** |
| NTC | 0.00 ± 0.00 | n/a | 0.00 ± 0.00 | n/a | 0.00 ± 0.00 | n/a |
| MTC | -0.60 ± 0.36 | 0.1038 | -0.68 ± 0.44 | 0.1106 | -0.55 ± 0.34 | 0.1041 |
| Scrambled Mimic | -1.26 ± 0.82 | 0.1163 | 3.10 ± 1.29 | 0.0535 | -2.90 ± 1.87 | 0.1149 |
| Scrambled Hairpin Inhibitor | -0.07± 0.43 | 0.7956 | 2.83 ± 1.16 | 0.0515 | -4.13 ± 2.03 | 0.0717 |

**^††^** Negative values denote down-regulation, while positive values denote up-regulation
